# 

**Published:** 1832-05

**Authors:** 


### MONTHLY LIST OF MEDICAL BOOKS.

[*Medical Works cannot be entered on this List unless a copy be sent for the purpose, the titles of Books having frequently been sent to us as published which have not been published for weeks, or even months, after.*]

Dissertatio Inauguralis Medica de Sanguinis in Periculosa Hæmorrhagia Uterina Transfusione. Auctore CAROLO WALLER, M.D., Societatis Medicæ Londinensis, Sodali, &c.—8vo. pp. 29. Erlangæ.

The Dissector's Guide, or Student's Companion. Illustrated by numerous Woodcuts, clearly exhibiting and explaining the Dissection of every part of the Human Body. By EDWARD WM. TUSON, F.L.S., Member of the Royal College of Surgeons in London, Lecturer on Anatomy and Physiology, &c.—12mo. pp. 219. Wilson, London.

*** This is a cheap and very useful guide for anatomical students: it is calculated especially to assist the progress of the beginner.

The Cyclopædia of Practical Medicine. Edited by JOHN FORBES, M.D., A. TWEEDIE, M.D., and JOHN CONOLLY, M.D. Part IV.—Royal 8vo. pp. 127. Sherwood and Co. London.

A Practical Treatise on Uterine Hemorrhage, in connexion with Pregnancy and Parturition. By JOHN T. INGLEBY, Member of the Royal College of Surgeons, one of the Surgeons to the General Dispensary, Surgeon to the Magdalen Asylum, and Lecturer on Midwifery at the School of Medicine in Birmingham.—8vo. pp. 276. Longman, London; Drake, Birmingham.

Lithotrity and Lithotomy compared: being an Analytical Examination of the present Methods of Treating Stone in the Bladder; with Suggestions for rendering Lithotrity applicable to the Disease in almost all its Stages and Varieties, and Remarks on the General Treatment of Gravel and Stone. By THOMAS KING, M.D., M.R.C.S., Surgeon to his Excellency the French Ambassador, Lecturer on Surgery, ex-Elève de Première Classe de l'Ecole Pratique, and formerly House-Surgeon to the Hôtel Dieu in Paris.—8vo. pp. 320; plates. Longman, London.

A Treatise on the Injuries, the Diseases, and the Distortions of the Spine; founded on an Essay to which the JACKSONIAN PRIZE for the year 1826 was adjudged by the Royal College of Surgeons. By R. A. STAFFORD, Member of the Royal College of Surgeons, Surgeon to the St. Marylebone Infirmary, and formerly House-Surgeon to St. Bartholomew's Hospital. —8vo. pp. 302. Longman, London.

Part VI. The Principles and Practice of Obstetric Medicine, in a Series of Systematic Dissertations on Midwifery, and on the Diseases of Women and Children. Illustrated by numerous Plates. By DAVID D. DAVIS, M.D., M.R.S.L., Professor of Midwifery in the University of London, &c.—4to. Taylor, London.

On the Nature and Cure of Glandular Diseases, especially those denominated Cancer, with the Mode of Treatment; and on the too frequent Use of Mercury; strongly recommended to the serious Consideration of every Individual: with a Detail of various Cases in which Cancer has been completely removed without the use of the Knife; illustrated by two Plates; also on Organic Affections of the Stomach, with a few Remarks on Cholera. And, in an Appendix, two Cases: 1, of Fissure of the Cranium; 2, of Preternatural Enlargement of the Heart. By Sir CHARLES ALDIS, Surgeon and Accoucheur, &c.—8vo. pp. 116. Highley, London

### APOTHECARIES' HALL.

**Names of Gentlemen** to whom the Court of Examiners have granted Certificates:

APRIL 5.

Benjamin Meredith Bradford, of Chepstow
Joseph Cunnington, King's Cliffe
Freeman Eaton, Grantham
Carsten Holthouse, Edmonton
John Stanton, Bristol
George Turner, Lofthouse
Edward Williams, Caerphilly

APRIL 12.

Matthew Brunskill, of Kirkby Stephen
Thomas Clarkson, West Welton, Yorkshire
Jallard Jennings Edenborough, London
Thomas Grundy, Warrington
Henry Hall, Ashmore, Dorsetshire
William Haine Maunder, Honiton
Richard Pullen, Leeds
Joseph Walker Ratcliffe, Wrexham
Henry Thomas, Halifax
John Whyte, Munkirk

APRIL 19.

Thomas Evans Brinsden, of Marlborough
Augustus Cooke, Yortworth
Richard Henry Crow, Wrotham
Benjamin Davies, London
David Hartley, Clifton
Thomas Edward Osmond, Thorp, Essex
George Ord, Newcastle
William Rayner, Lincoln
William Richardson, London
John Skevington, Ashbourn

APRIL 26.

John Bartlett, of Great Bedwin
William Robert Clavey, Bath
Richard Ebsworth, Shillingford
George Ellerton, Kippax
John Freeland Fergus, Newcastle on Tyne
Edward Hodges, Leicester
John Jobson, Newcastle on Tyne
George Mousley, Chilcote
Samuel Richards, London
Zachariah Robert Stewart, Southwark
Joseph Sadler, Barnsley.

TO CORRESPONDENTS.

*We have more than once been indebted to the author of the paper " on Erysipelas," of L— C— street, for some interesting Communications. That which we now acknowledge, however, is too inaccurately and carelessly written to justify us in giving it to the profession.*

